# Supplementary material for: Characterizing cell-type spatial relationships across length scales in spatially resolved omics data
Source: Nat Commun. 2025 Jan 3;16:350. doi: 10.1038/s41467-024-55700-1 (PMC11699133; doi:10.1038/s41467-024-55700-1)
Supplement: Supplementary file 3 — Supplementary Software 1 [file 41467_2024_55700_MOESM3_ESM.zip › CRAWDAD/plots/supplementary/S7_cluster_exp_NGPL_annot.pdf]

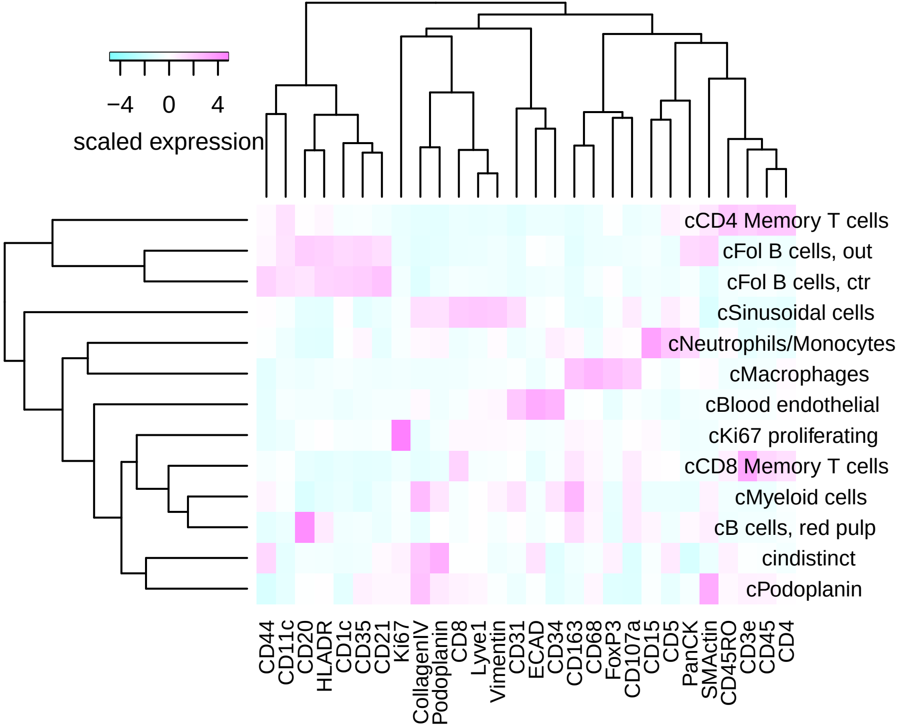

−4
0
4
scaled expression
cCD4 Memory T cells
cFol B cells, out
cFol B cells, ctr
cSinusoidal cells
cNeutrophils/Monocytes
cMacrophages
cBlood endothelial
cKi67 proliferating
cCD8 Memory T cells
cMyeloid cells
cB cells, red pulp
cindistinct
cPodoplanin
CD44
CD11c
CD20
HLADR
CD1c
CD35
CD21
Ki67
CollagenIV
Podoplanin
CD8
Lyve1
Vimentin
CD31
ECAD
CD34
CD163
CD68
FoxP3
CD107a
CD15
CD5
PanCK
SMActin
CD45RO
CD3e
CD45
CD4
